# Immunohistochemical expression of estrogen and progesterone receptors in endometrial polyps: A comparison between benign and malignant polyps in postmenopausal patients

**DOI:** 10.3892/ol.2014.2004

**Published:** 2014-03-28

**Authors:** ARMANDO ANTUNES, JOSÉ VASSALLO, ANDERSON PINHEIRO, ROGÉRIO LEÃO, AARÃO MENDES PINTO NETO, LUCIA COSTA-PAIVA

**Affiliations:** 1 Department of Obstetrics and Gynecology, State University of Campinas School of Medicine, Campinas, São Paulo 13.083-881, Brazil; 2 Department of Anatomical Pathology, State University of Campinas School of Medicine, Campinas, São Paulo 13.083-881, Brazil

**Keywords:** endometrial polyp, postmenopausal, estrogen receptor, progesterone receptor

## Abstract

The aim of the present study was to evaluate estrogen receptor (ER) and progesterone receptor (PR) expression in the glandular epithelium and stroma of benign and malignant endometrial polyps of postmenopausal patients. A total of 1,050 females underwent surgical hysteroscopy at the Professor Dr José Aristodemo Pinotti Women’s Hospital, Center for Integral Attention to Women’s Health of the State University of Campinas, between January 1998 and December 2008. Of the total number, 390 postmenopausal females with endometrial polyps were included in the study. Polypoid lesions were histologically classified as benign lesions (endometrial polyps and polyps with non-atypical simple hyperplasia or non-atypical complex hyperplasia) and premalignant and malignant lesions (polyps with atypical simple hyperplasia or atypical complex hyperplasia and carcinomatous polyps). ER and PR expression was evaluated by immunohistochemistry according to cell staining, intensity of nuclear staining and final score. The final score for receptor expression was compared between the benign and premalignant/malignant polyps. The prevalence of malignancy in endometrial polyps was 7.1% and was associated with postmenopausal bleeding. Only the final score for ER expression in the stroma of endometrial polyps was higher in the benign group than in the premalignant/malignant group, and this difference was significant. However, no difference was identified in PR expression. In addition, the risk of malignancy in endometrial polyps was significantly higher when the expression of ER and PR was negative in the stromal component of the polyp (P<0.01). The malignancy of endometrial polyps was also associated with a low expression of stromal ER, however, PR expression did not show any association with the risk of malignancy.

## Introduction

Endometrial polyps are localized overgrowths of the endometrium, with histological features composed of the irregular proliferation of glands and stroma, containing thick-walled blood vessels and lined by pseudostratified or flat epithelium (1).

The prevalence of polyps ranges between 7.8 and 34.9%, depending on the method used for diagnosis and the study population (2). Prevalence has been found to increase with age and is higher in postmenopausal patients compared with premenopausal patients (3).

The malignancy rate associated with endometrial polyps is low, and in a recent meta-analysis on the oncogenic potential of polyps, it was observed that the malignancy rate of endometrial polyps ranged between 0.8 and 8% in the different studies analyzed (4). In our previous study, a higher occurrence of premalignant and malignant polyps was observed in postmenopausal females aged over 60 years with vaginal bleeding (5). Other studies have also shown an association between malignancy and certain risk factors, including obesity, arterial hypertension, diabetes mellitus and tamoxifen use (4,6).

Hormonal factors appear to be present in the pathogenesis of endometrial polyps, and estrogen and progesterone are known modulators of endometrial proliferation and differentiation by means of steroid receptors. Furthermore, the development of polyps may be associated with higher receptor expression in the glandular epithelium, which subsequently leads to focal hyperplasia of the endometrium (7). Few studies with a limited number of tissue samples have assessed the expression of these receptors in endometrial polyps (8–10). In the glandular epithelium of endometrial polyps, the immunohistochemical expression of the estrogen receptor (ER) and progesterone receptor (PR) is higher than that in the adjacent endometrium. However, in the stromal component of the endometrial polyps, only ER expression is higher than in the adjacent endometrium. The same is not observed with PR (8).

Despite a low prevalence of malignancy in endometrial polyps, the role of ER and PR expression in the mechanisms of carcinogenesis remains unknown. No data evaluating these receptors in malignant polyps exists in the literature and therefore, we hypothesized that there may be a difference in the receptor expression between malignant and benign polyps.

The aim of the present study was to evaluate ER and PR expression in the glandular epithelium and stroma of malignant and benign polyps in postmenopausal patients.

## Materials and methods

### Patients

The present study was conducted at the Professor Dr. José Aristodemo Pinotti Women’s Hospital (Center for Integral Attention to Women’s Health) of the State University of Campinas (UNICAMP; Campinas, Brazil). Approval was obtained from the Research Ethics Committee of the UNICAMP School of Medicine (769/2009), additionally the ethics committee waived the requirement for patient consent. According to information stored in the computerized database of this institution, 6,018 surgical hysteroscopies were performed between January 1998 and December 2008 for the diagnosis and treatment of diverse uterine conditions. Of the females examined, 1,050 underwent surgical treatment of endometrial polyps and of these, 508 were postmenopausal. Females with no histological confirmation of endometrial polyps and users of hormonal therapy and tamoxifen were excluded. As a result, 390 postmenopausal females, aged between 39 and 86 years and diagnosed with endometrial polyps determined by ultrasound or diagnostic hysteroscopy were included in this study. Menopause was defined as amenorrhea that had lasted for >12 months.

Clinical, histopathological and hysteroscopic data were retrieved from patient medical records and the following clinical characteristics were observed: Age, postmenopausal bleeding, time since menopause, parity, presence of arterial hypertension, obesity, diabetes mellitus and history of breast cancer.

Diagnostic hysteroscopy was performed using a 2.8-mm optical system (Karl Storz GmbH and Co., KG, Tuttlingen, Germany), and for distension of the uterine cavity, a CO_2_ and saline infusion was used. Surgical hysteroscopy was performed by a gynecologist with the patient under spinal anesthesia, and a 10-mm resectoscope with a loop electrode was used for the surgical procedure (Karl Storz GmbH and Co., KG). Distension of the uterine cavity was obtained by administration of the 1.5% glycine solution, prior to the evaluation of the endocervical channel and endometrial cavity. Resection of the endometrial polyps was performed by electrocautery using the monopolar mode of energy.

Pathologists from the Department of Pathological Anatomy of the UNICAMP Medical School analyzed the endometrial samples obtained, using hematoxylin and eosin (H&E) staining. Polyps were then classified as benign, non-atypical simple hyperplasia, non-atypical complex hyperplasia, atypical simple hyperplasia, atypical complex hyperplasia or malignant.

### Construction of tissue microarray (TMA)

Initially, a pathologist from the Department of Pathological Anatomy of the UNICAMP School of Medicine studied the slides representative of endometrial polyps stained with H&E. The two regions that best represented the stroma and glandular epithelium were then selected for the construction of the TMA following a technique validated for the endometrium (11). Subsequently, the selected regions were identified in archival paraffin blocks (donor blocks). These marked donor blocks were sent to the Laboratory of Immunohistochemistry of the Division of Pathologic Anatomy of the A.C. Camargo Cancer Center (São Paulo, Brazil) for the construction of the receptor blocks using the TMA technique. A TMA (Beecher Instruments Inc., Silver Springs, MD, USA), available at the Department of Pathologic Anatomy at the A.C. Camargo Cancer Center, was used. Cylinder cores measuring 1.0 mm from the region of interest, which were obtained by the previously described equipment, were transferred to a new block with a two-dimensional layout and 0.2 mm spacing between the cores, then determined and recorded. From this new block, termed the recipient TMA block, histological sections were obtained using a manual microtome and transferred by adhesive tape to special adhesive-coated slides (Instrumentics, Inc., Hackensack, NJ, USA). The adhesive tape was then removed under exposure to ultraviolet light. Next, the sections were stored, paraffin-embedded, vacuum-packed and frozen at −20°C.

### Immunohistochemistry

ER and PR expression was evaluated at the Laboratory of Immunohistochemistry of the Department of Pathology of the A.C.Camargo Cancer Center. The TMA sections were 5-μm thick and deparaffinization was performed for 24 h at 60°C in an incubator. Subsequently, the sections were rinsed in xylene at 60°C for 20 min and at room temperature for 20 min, followed by rinsing with 100% ethanol for 30 sec, 85% ethanol for 30 sec and 70% ethanol for 30 sec. The sections were then washed under distilled running water.

A 10 mM citrate buffer solution (pH 6.0) was heated to boiling point in a pressure cooker without sealing the lid (Eterna^®^; Nigro Aluminium Ltd., São Paulo, Brazil). The slides were then immersed and the lid was sealed with the safety valve in open position. Following the release of the saturated vapor, the safety valve was lowered until total pressurization was reached. The timing was started once the pressure indicator valve had reached the maximum point (~4 min). The pressure cooker remained closed under running water until total depressurization. The lid of the cooker containing the slides was then opened and the slides were washed in distilled running water.

Endogenous peroxidase was blocked with 3% hydrogen peroxide [H_2_O_2_ (10 vol)] with three changes of 10 min each. The sections were then washed in distilled running water and 10 mM phosphate-buffered saline [PBS (pH 7.4)] for 5 min.

Next, the slides were incubated with primary antibody diluted in a predefined titer in PBS buffer containing 1% bovine serum albumin (A9647; Sigma-Aldrich, St. Louis, MO, USA) and 0.1% sodium azide for 18 h in a humidity chamber at 4°C. The procedure used primary monoclonal antibodies against ER (M7047; clone 1D5; 1:250) and PR (M3569; clone PgR 636; 1:500) (Dako^®^, Carpinteria, CA, USA).

The slides were washed with three changes of PBS buffer for 3 min each and incubated for 30 min at 37°C with the Advance™ horseradish peroxidase (HRP)-linked secondary antibody (K4068; Dako). The slides were washed again with three changes of PBS buffer for 3 min each and then incubated with the Advance HRP enzyme for 30 min at 37°C. Following a final washing with three changes of PBS buffer for 3 min each, the slides were incubated in the following substrate solution: 100 mg 3,3′-diaminobenzidine-tetrahydrochloride (D-5637; Sigma-Aldrich), 1 ml dimethyl sulfoxide, 1 ml 6% H_2_O_2_ (20 vol) and 100 ml PBS for 5 min at 37°C, protected from light. Next, the slides were washed in distilled running water for 3 min and counterstained with Harris’ hematoxylin for 1 min, followed by a final thorough washing in distilled running water. The slides were then immersed twice into ammoniacal water (0.5% ammonium hydroxide solution) and washed in distilled running water. The sections were dehydrated in the following solutions: 80% ethanol for 30 sec, 95% ethanol for 30 sec, 100% ethanol twice for 30 sec each and xylene four times for 30 sec each. The slides were then mounted on Entellan neu (1.07961; Merck, Darmstadt, Germany) and on microscopy, a final reaction product was observed as a golden brown precipitate, varying according to the type of marker.

### Immunohistochemical analysis

#### Hormonal receptors (ER and PR)

**Figure 1:**
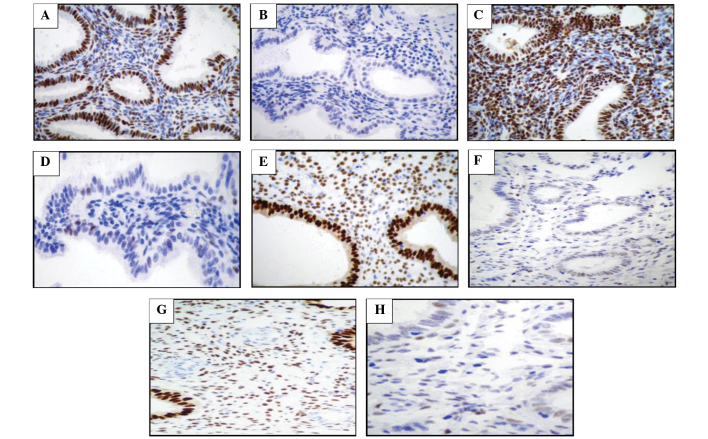
Representative immunohistochemical nuclear staining of ER and PR. (A) ER^+^ and (B) ER^−^ in the glandular epithelium, (C) ER^+^ and (D) ER^−^ in the stroma, (E) PR^+^ and (F) PR^−^ in the glandular epithelium and (G) PR^+^ and (H) PR^−^ in the stroma. ER, estrogen receptor; PR, progesterone receptor.

The TMA slides were read manually by only one pathologist using conventional light microscopy (Zeiss AxioPhot microscope, Carl Zeiss Microscopy, LLC, Thornwood, NY, USA). ER and PR expression was evaluated in the stroma and glandular epithelium of the polyp tissues using a semi-quantitative method of nuclear reaction through analysis of the percentage of stained cells, the intensity of nuclear staining and the final score (12). The percentage of stained cells was visually estimated and categorized as follows: Grade 0, no staining; grade 1, <1% staining; grade 2, 1–10% staining; grade 3, 11–33% staining; grade 4, 34–66% staining; and grade 5, >66% staining. With regard to the intensity of nuclear staining, the staining was categorized as follows: Grade 0, negative; grade 1, weak reaction; grade 2, moderate reaction; and grade 3, intense reaction (12). The sum of positivity and intensity resulted in a final score that ranged between 0 and 8 (excluding value 1) (Fig. 1).

### Statistical analysis

For statistical analysis, the polyps were grouped as benign (including polyps of the endometrial mucosa and polyps with non-atypical simple hyperplasia or non-atypical complex hyperplasia) or premalignant/malignant (including polyps with atypical simple hyperplasia or atypical complex hyperplasia and carcinomatous polyps). Clinical characteristics between the groups of benign and malignant polyps were compared using the χ^2^, Fisher’s exact or Mann-Whitney non-parametric tests. To compare the final scores of ER and PR expression in the glandular epithelium and stroma of the polyps, a final score of ≤2 was considered a negative reaction and a final score of ≥3 was considered a positive reaction. This comparison was made using Fisher’s exact test and the χ^2^ test. A combination of ER/PR expression in the glandular epithelium and stroma of the endometrial polyps in comparison to the malignant and benign lesions was calculated using Fisher’s exact test. The Statistical Analysis System program, version 9.2 (SAS Institute Inc., Cary, NC, USA) was used for these calculations. P<0.05 was considered to indicate a statistically significant difference.

## Results

### Histological diagnosis

Table I shows the histological diagnosis of resected lesions. In total, 362 benign lesions were diagnosed (92.82%), including 313 endometrial (80.26%), 41 non-atypical simple hyperplasia (10.5%) and eight non-atypical complex hyperplasia (2.05%) polyps. The premalignant lesions consisted of five polyps with atypical simple hyperplasia (1.28%) and three polyps with atypical complex hyperplasia (0.76%). In addition, 20 malignant polyps (5.11%) were diagnosed. Among the malignant polyps, endometrioid adenocarcinoma was the histological type of the majority. However, one case with less-differentiated endometrial carcinoma and an additional case with serous endometrial cancer were observed.

### Clinical characteristics of postmenopausal patients

The mean age of the females with benign polyps was 61.7±7.8 years (mean ± standard deviation) and 64.4±10.4 years for those with malignant polyps. No significant difference was identified in the mean age at menopause between the two groups (48.9±7.5 vs. 50.4±4.8; P=0.236). Table II shows a comparison of the clinical characteristics of the patients studied. No differences associated with the presence of comorbid disorders, including arterial hypertension, diabetes mellitus, breast cancer, obesity and parity, were identified among the females with benign and premalignant/malignant polyps. However, the presence of postmenopausal bleeding was significantly greater in females with premalignant/malignant polyps (P=0.0015; Table II).

### ER and PR expression

By comparing the final ER and PR score between the benign and premalignant/malignant polyps, only the final score of ER expression in the stroma of the endometrial polyps was observed to be higher in the benign polyps compared with the premalignant and malignant polyps, and showed a statistically significant difference (Table III).

In addition, by comparing the combined expression of ER/PR, the risk of malignancy in the polyps was observed to be significantly higher when the expression of the two receptors was negative (ER^−^/PR^−^) in the stroma of the endometrial polyps (odds ratio, 6.5; 95% confidence interval, 2.05–20.29). However, no significant difference was identified in the glandular epithelium (Table IV).

## Discussion

The present study was conducted to evaluate ER and PR expression in malignant and benign endometrial polyps of postmenopausal patients. The results indicated that malignant polyps exhibit a lower glandular and stromal ER expression than benign polyps, however, PR expression was not found to correlate with malignancy.

To the best of our knowledge, the present study concerning ER and PR expression in postmenopausal patients is the largest case study to have evaluated an association with malignancy. The prevalence of malignancy in the sample studied was 7.1%, and postmenopausal bleeding was the only clinical parameter found to correlate with the risk of malignancy in the endometrial polyps. In our previous study, a prevalence of 4.1% (13) was identified. These prevalence rates are consistent with those identified in other studies, which have shown a prevalence of malignancy ranging between 0.8 and 8.0% (14,15).

With regard to hormone receptor expression, the ER and PR are specific nuclear receptors that belong to the steroid receptor family. The activity of the ER is based on specific regions of the gene and furthermore, the formation and concentration of new receptors appear to be self-regulated and dependent on hormonal factors (16). However, for progesterone, the tissue expression of the PR has not been found to correlate with the hormonal status found in postmenopausal patients, in which progestational activity is not observed. In addition, the induction of PR formation in the endometrium is mainly a consequence of estrogen stimulation (17).

In the present study, the benign and premalignant/malignant polyps were observed to exhibit a higher expression of ER and PR in glandular cells than in stromal cells. This higher glandular expression of the ER and PR has also been observed in postmenopausal endometrial polyps when compared with the atrophic endometrium (9) or adjacent endometrium (7,8,18). Other studies have also demonstrated that polyps in postmenopausal females exhibit increased ER expression in the stroma and glandular epithelium compared with polyps in premenopausal females (10,19). However, PR expression is higher only in the glandular epithelium, with no difference in expression identified in the stroma (19).

Few previous studies have investigated the pathogenesis of endometrial polyps in detail, however, the present study is the first to compare the ER and PR expression between benign and malignant cases. The benign polyps were found to show higher ER expression in the glandular epithelium and stroma. However, no difference was identified with regard to PR when compared with the premalignant/malignant polyps. This appears to indicate that benign polyps in postmenopausal patients may respond to an increased number of receptors, as a consequence of low estrogen levels during the menopause. In addition, high expression in the glandular epithelia indicates a higher sensitivity of these structures to steroid hormones, which may be responsible for the development of benign polyps in the presence of low serum estrogen levels, while malignant polyps appear to be developed by a different etiology.

In contrast to the high ER and PR expression observed in benign endometrial polyps, one study has demonstrated that the loss of steroid receptors is an early event in endometrial carcinogenesis, and that endometrial carcinoma usually exhibits a lower level of ER and PR than the normal endometrium or in endometrial hyperplasia (20). These observations indicate that the development of benign and carcinomatous polyps may follow distinct pathways. By contrast, the majority of studies show that estrogen promotes endometrial carcinogenesis directly by stimulating the rapid proliferation of epithelial cells. In addition, high ER expression is observed in hyperplasia and carcinoma in populations of stromal and epithelial cells (21–23).

According to the literature, ER and PR expression may be lower in more advanced tumors and less-differentiated tumors, which is a factor of worse prognosis (22,23). In the present study, the presence of serous carcinoma and less-differentiated carcinoma may have contributed to a decrease in ER expression in the group of premalignant/malignant polyps. In addition, when the expression of the two receptors (ER/PR) was negative a malignancy risk that was six times higher was observed. Other studies have also demonstrated that steroid receptor expression is not an independent prognostic factor for endometrial cancer, and uncertainty remains as to the usefulness of determining receptor expression in patients with endometrial neoplasms (24,25). No previous study has evaluated these receptors in malignant polyps for the comparison of results. However, it may be inferred from these differences that the pathways of carcinogenesis in endometrial polyps may be different from those observed in endometrial cancer or may be similar to neoplasms of worse prognosis.

Discrepancies with regard to the results of the present study and the various studies in the literature may in part be explained by variations in methodology, particularly differences in the antibody specificity and dilutions used. The lack of consensus in the criteria defined for the positivity and semi-quantitative nature of the method may have also contributed to the different results between studies, according to the criteria used. An additional possible limitation is the small number of premalignant/malignant polyps analyzed in the present case study, which may have interfered with the capacity of statistical tests to identify significant differences between the groups. However, it is important to highlight the fact that the number of premalignant/malignant polyps may be high, in view of the low prevalence of malignancy associated with polyps.

In conclusion, the observations of the present study have shown that polyps in postmenopausal patients have high ER expression in the stroma and glandular epithelium. However, this expression is lower in premalignant/malignant polyps compared with benign polyps. These results indicate that lower ER expression may be one more risk factor for the malignancy potential of polyps in postmenopausal females. Polypectomy has been routinely indicated to stop bleeding and to exclude malignancy. No tool is currently available to make predictions of the malignancy of these lesions, and histological evaluation of the resected polyp continues to be the only form of diagnosing malignant cases. The usefulness of measuring receptors in polyps remains questionable. The real etiology of polyps and their mechanisms of carcinogenesis appear to occur by different mechanisms that are currently unclear, but remain necessary for the adequate management of endometrial polyps.

## Figures and Tables

**Table I:** Histological diagnosis of endometrial polyps in postmenopausal patients.

Histological diagnosis	n	%
Benign		
Endometrial polyp	313	80.25
Polyp without atypical simple hyperplasia	41	10.51
Polyp without atypical complex hyperplasia	8	2.05
Subtotal	362	92.82
Premalignant/malignant		
Polyp with atypical simple hyperplasia	5	1.28
Polyp with atypical complex hyperplasia	3	0.76
Polyp with endometrioid adenocarcinoma	18	4.61
Polyp with less-differentiated endometrial carcinoma	1	0.25
Polyp with serous endometrial cancer	1	0.25
Subtotal	28	7.17
Total	390	100

**Table II:** Clinical characteristics of postmenopausal patients with benign and malignant endometrial polyps, and the prevalence of malignancy (n=390).

	Benign		Premalignant/malignant		
			
Characteristics	n	%	n	%	P-value
Age, years					0.8549^a^
<40	1	100.0	0	0.0	
40–59	153	93.3	11	6.7	
≥60	207	92.4	17	7.6	
Postmenopausal bleeding					0.0015^b^,^c^
Yes	143	87.7	20	12.3	
No	210	96.3	8	3.7	
Subarachnoid hemorrhage					0.1847^b^
Yes	254	91.7	23	8.3	
No	107	95.5	5	4.5	
Diabetes mellitus					0.2323^b^
Yes	103	90.4	11	9.6	
No	257	93.8	17	6.2	
Breast cancer					0.6661^a^
Yes	20	90.9	2	9.1	
No	341	92.9	26	7.1	
Body mass index					0.0721^b^
<30	150	95.5	7	4.5	
≥30	204	90.7	21	9.3	
Parity					0.7098^a^
Nulliparous	28	96.6	1	3.4	
Multiparous	331	92.5	27	7.5	

a Fisher’s exact and

b χ^2^ tests;

c Statistically significant.

Some patient data was missing from the patient records and therefore the columns may not add up to 390.

**Table III:** Final ER/PR score in benign and malignant polyps of postmenopausal patients (n=390).

Final score	Benign (n=362), %	Premalignant/malignant (n=28), %	P-value	OR (95% CI)
ER gland (n=381)			0.5721^a^	
Positive	85.6	81.5		1.0
Negative	14.4	18.5		1.4 (0.49–3.73)
ER stroma (n=384)			0.0024^a^,^b^	
Positive	82.9	59.3		1.0
Negative	17.1	40.7		3.3 (1.48–7.54)^b^
PR gland (n=379)			0.7089^c^	
Positive	93.4	92.9		1.0
Negative	6.6	7.1		1.1 (0.24–4.91)
PR stroma (n=381)			0.1004^c^	
Positive	89.8	77.8		1.0
Negatives	10.2	22.2		1.4 (0.56–3.30)

a Fisher’s exact and

c χ^2^ tests;

b Statistically significant.

ER, estrogen receptor; PR, progesterone receptor; OR, odds ratio; CI, confidence interval.

**Table IV:** Comparison of the combined final ER/PR score in benign and malignant polyps of postmenopausal patients (n=390).

Final ER/PR score	Benign (n=362), %	Premalignant/malignant (n=28), %	P-value^a^	OR (95% CI)
ER/PR gland (n=372)			0.2269	
ER^+^/PR^+^	96.3	90.9		1.0
ER^+^/PR^−^	3.7	9.1		2.6 (0.54–12.58)
ER^−^/PR^+^	76.6	100.0		2.0 (0.70–5.64)
ER^−^/PR^−^	23.4	0.0		0.6 (0.03–10.72)
ER/PR stroma (n=375)			0.0055^b^	
ER^+^/PR^+^	93.1	93.3		1.0
ER^+^/PR^−^	6.9	6.7		1.0 (0.12–7.74)
ER^−^/PR^+^	74.1	54.5		2.7 (0.98–7.41)
ER^−^/PR^−^	25.9	45.5		6.5 (2.05–20.29)

a Fisher’s exact test.

b Statistically significant.

ER, estrogen receptor; PR, progesterone receptor; OR, odds ratio; CI, confidence interval.
